# Comment on: “Gender-based comparison of clinical, epidemiological, and laboratory characteristics in hemodialysis patients: a retrospective analysis”

**DOI:** 10.1590/1806-9282.20260520

**Published:** 2026-08-07

**Authors:** Ömer Faruk Rahman, Alper Özbakkaloğlu

**Affiliations:** 1 İzmir Bakırçay University, Department of Cardiovascular Surgery – İzmir, Turkey.

Dear Editor,

We read with great interest the article by Oliveira et al.^1^ entitled “Gender-based comparison of clinical, epidemiological, and laboratory characteristics in hemodialysis patients: a retrospective analysis.” While the study provides valuable insights into sex-related differences in chronic kidney disease, we believe that several methodological aspects may influence the interpretation of the findings.

First, the conditions under which laboratory parameters were obtained were not clearly specified. In particular, it remains unclear whether blood samples were collected in the fasting state and whether measurements were performed before or after hemodialysis sessions. Given that parameters such as potassium, phosphorus, urea, and lipid levels are significantly affected by these factors, the lack of standardization may represent an important source of bias in intergroup comparisons^2,3^. Furthermore, previous studies have shown that hemodialysis and ultrafiltration can significantly influence serum lipid levels, and measurements obtained at the beginning of a dialysis session may underestimate true lipid values^4^. In this context, the absence of dialysis-related parameters such as dialysis adequacy, ultrafiltration volume, session frequency, and sampling timing may substantially limit the interpretation of lipid-related findings. Therefore, the observed sex differences may, at least in part, be attributable to dialysis-related factors.

Second, although the inclusion of both chronic kidney disease and acute kidney injury or acute-on-chronic kidney disease patients was acknowledged by the authors as a limitation, the resulting heterogeneity may not be limited to this aspect alone. Among patients with chronic kidney disease, factors such as disease duration, dialysis vintage, and session frequency may also influence clinical and biochemical parameters. These factors are known to affect mineral metabolism and electrolyte levels and may have contributed to the observed differences^5^. Therefore, not only the combined evaluation of acute and chronic conditions but also the effects of disease duration and dialysis duration should be considered when interpreting the findings.

Third, medication use was assessed only as present or absent, without consideration of dose, treatment duration, or adherence. Although this limitation was noted by the authors, it may still represent an important factor influencing the interpretation of the effects of statins and renin-angiotensin-aldo-sterone system blockers.

In addition, the borderline statistical significance observed in statin use between sexes (p=0.052) is noteworthy. This finding may reflect limited statistical power due to the relatively small sample size and should therefore be interpreted with caution.

In conclusion, while the study provides important findings, consideration of the above methodological issues may contribute to a more accurate interpretation of the results.

Sincerely,

## Data Availability

The datasets generated and/or analyzed during the current study are available from the corresponding author upon reasonable request.
